# Transcriptional analysis identifies potential novel biomarkers associated with successful ex‐vivo perfusion of human donor lungs

**DOI:** 10.1111/ctr.14570

**Published:** 2022-01-10

**Authors:** John Robert Ferdinand, Morvern Isabel Morrison, Anders Andreasson, Catriona Charlton, Alisha Kaur Chhatwal, William Earl Scott, Lee Anthony Borthwick, Menna Ruth Clatworthy, Andrew J. Fisher

**Affiliations:** ^1^ Molecular Immunity Unit University of Cambridge, Department of Medicine Cambridge UK; ^2^ Newcastle University Translational and Clinical Research Institute Faculty of Medical Sciences, Newcastle University Newcastle Upon Tyne UK; ^3^ Institute of Transplantation Freeman Hospital Newcastle Upon Tyne UK; ^4^ Cellular Genetics Wellcome Sanger Institute Hinxton UK

**Keywords:** artificial organs, chemokine receptors, chemokines, clinical trial, support devices: lung

## Abstract

**Background:**

Transplantation is an effective treatment for end‐stage lung disease, but the donor organ shortage is a major problem. Ex‐vivo lung perfusion (EVLP) of extended criteria organs enables functional assessment to facilitate clinical decision‐making around utilization, but the molecular processes occurring during EVLP, and how they differ between more or less viable lungs, remain to be determined.

**Methods:**

We used RNA sequencing of lung tissue to delineate changes in gene expression occurring in 10 donor lungs undergoing EVLP and compare lungs that were deemed non‐transplantable (*n* = 4) to those deemed transplantable (*n* = 6) following perfusion.

**Results:**

We found that lungs deemed unsuitable for transplantation had increased induction of innate immune pathways and lower expression of oxidative phosphorylation related genes. Furthermore, the expression of SCGB1A1, a gene encoding an anti‐inflammatory secretoglobin CC10, and other club cell genes was significantly decreased in non‐transplantable lungs, while CHIT‐1 was increased. Using a larger validation cohort (*n* = 17), we confirmed that the ratio of CHIT1 and SCGB1A1 protein levels in lung perfusate have potential utility to distinguish transplantable from non‐transplantable lungs (AUC .81).

**Conclusions:**

Together, our data identify novel biomarkers that may assist with pre‐transplant lung assessment, as well as pathways that may be amenable to therapeutic intervention during EVLPAQ6.

## 1 INTRODUCTION

Lung transplantation is an effective treatment for selected patients with life‐threatening end‐stage lung disease. Unfortunately, a shortage of donor organs limits access to transplantation for many patients who might benefit from this treatment.^1^ One strategy to address this challenge is to utilize lungs from extended criteria donors (ECD) with advanced donor age, a smoking history, reduced lung function or co‐morbidities.^2,^, ^3^ ECD lungs potentially have an increased risk of poor outcomes if sub‐optimal organs are inadvertently used.^4,^, ^5^ *Ex‐vivo* lung perfusion (EVLP) provides an opportunity to functionally assess, and potentially recondition, lungs deemed unsuitable for immediate transplantation, to aid organ utilization decisions and increase the donor lung pool.^6,^, ^7^ The decision to accept organs for transplantation after EVLP is currently based on visual appearance and physiological parameters such as oxygenation, lung compliance, pulmonary vascular resistance, and peak airway pressure. However, reported discard rates of perfused lungs are highly variable, ranging from 10 to 60%, suggesting that some donor lungs are being inappropriately used and others unnecessarily declined for transplant.^8^

The cellular and molecular events occurring during EVLP have not been well characterized, and how these processes differ between lungs that are deemed useable compared with those that are discarded is unknown. In addition, there is a pressing need to identify novel predictive biomarkers that can be utilized during EVLP. Cellular injury during lung retrieval and storage results in the release of danger signals that activate immune cells, so‐called sterile inflammation, causing further collateral tissue damage.^9^ We previously demonstrated the feasibility of measuring pro‐inflammatory and tissue injury signals in EVLP perfusion fluid to identify unsuitable donor lungs.^10^ Furthermore, we recently demonstrated that interleukin (IL)‐1β and tumor necrosis factor (TNF) levels in perfusate after 30 min of EVLP are predictive of in‐hospital mortality post‐transplantation.^10^ Although these candidate proteins in perfusate may have potential utility as biomarkers, their selection is based on published literature that identified candidate proteins associated with primary lung graft dysfunction^11,^, ^12^ and does not provide a comprehensive means of assessing the molecular pathways impacted by EVLP nor of identifying novel biomarkers.

Here we utilized RNA‐sequencing (RNASeq) to generate an unbiased profile of the transcriptome of human donor lungs prior to, and following EVLP, comparing changes in gene expression between lungs deemed suitable for transplantation, and those non‐transplantable on the basis of standard physiological parameters. Overall, during EVLP, we observed an increase in a variety of immune pathway genes. When comparing lungs deemed non‐transplantable to transplantable, we found that the expression level of immune pathway genes, including NLRP3 inflammasome‐associated genes, increased to a greater extent in non‐transplantable lungs during EVLP as did expression of *CHIT1* (encoding chitotriosidase). In contrast the expression of genes involved in energy generation through oxidative phosphorylation (OXPHOS) were not increased in lungs during EVLP in non‐transplantable lungs in contrast to transplantable lungs. Furthermore, the expression of *SCGB1A1*, an anti‐inflammatory secretoglobin, and other club cell genes, were also significantly increased in transplantable lungs.

Overall, our data suggest that lungs deemed suitable for transplantation following EVLP have reduced induction of multiple immune pathways, are better able to generate ATP and may have increased club cell number or activation than those that are deemed non‐transplantable. Our study identifies specific outcome‐associated biomarkers and pathways amenable to therapeutic intervention during EVLP that will inform the design of future clinical trials in this area.

## 2 METHODS

Further methods are given in the supplement.

### 2.1 Study subjects and protocol

In this study, we utilized samples of whole donor lung tissue collected from a large cohort of highly characterized clinical EVLP procedures performed as part of the DEVELOP‐UK multicenter trial, which involved all five UK transplant centres.^13^ DEVELOP‐UK included 53 adult donor lungs which were deemed unsuitable for lung transplantation but met pre‐defined criteria for EVLP. Assessments were performed using a Vivoline LS1 EVLP circuit (Vivoline Medical AB, Lund, Sweden) following the Lund protocol with cellular perfusate (hematocrit 10–15%) and full flow perfusion of 100% donor calculated cardiac output. A full report of the outcomes of the DEVELOP‐UK study have been previously published.^13^ Samples were selected for the study presented here based on the availability of samples and the successful extraction of good quality RNA.

### 2.2 Sample collection

For collection of tissue for RNASeq a standardized sampling protocol was followed to allow collection from either the right middle lobe or lingula both before and after the EVLP assessment using a GIA surgical stapler (Medtronic, UK). Biopsies were snap frozen in liquid nitrogen as soon as possible after collection for subsequent RNA isolation. Samples were shipped frozen to Newcastle university for storage and from the individual trial centers and subsequently to the University of Cambridge for RNASeq analysis remaining frozen at all times to maintain RNA integrity.

To enable protein analysis of the perfusate a control sample was collected from the primed EVLP circuit before donor lung perfusion started. Repeated perfusate samples (5 ml) were then collected at 15 and 30 min after perfusion commenced and every 30 min thereafter until the end of perfusion. The perfusate samples were centrifuged at 180 g for 6 min at 4°C to remove cellular debris and then aliquoted into tubes before being frozen initially at −20°C and then transferred to a −80°C for longer term storage and subsequent laboratory analysis.

### 2.3 RNA extraction

RNA was extracted from small pieces of tissue snap frozen at the indicated time points. Samples were lysed in Lysis buffer (Invitrogen) using a precellys homogenizer (Bertin Instruments), RNA extracted using a Pure link RNA mini kit (Invitrogen) and contaminating DNA removed using Turbo DNase kit (Invitrogen). RNA was quantified using by absorbance and 280 nm (Nanodrop spectrophotometer) and quality assessed using a 2100 bioanalyzer (Agilent) with a RNA nano chip (Agilent).

### 2.4 RNASeq

RNAseq Libraries were made using TruSeq Stranded Total RNA library prep kit (Illumina) with 7 min fragmentation as per manufacturers instructions. Final libraries were amplified for 14 cycles by PCR and the final size assessed using a 2100 bioanalyzer (Agilent) with a HS DNA chip (Agilent). Pooling of finally libraries and sequencing was carried out by Eurofins on a Hiseq 2500 on a Rapid run 1 × 50.

### 2.5 RNASeq analysis

BCL files were demultiplexed using CASAVA, fastqs aligned to hg38 using hisat2, and counts determined using Featurecounts. Differential expression was carried out using DESeq2.

## 3 RESULTS

### 3.1 Transcriptomic analysis of lungs pre and post EVLP reveals induction of immune pathway genes

Between April 2012 and July 2014, 53 pairs of human donor lungs deemed unsuitable for immediate transplantation underwent EVLP in the DEVELOP‐UK clinical trial.^13^ Pre‐ and post‐EVLP samples were available from *n* = 10 donor lungs, of which *n* = 6 “Passed” EVLP assessment and were deemed transplantable, and *n* = 4 “Failed” assessment and were deemed non‐transplantable based on trial protocol criteria^13^ (Table 1).

**TABLE 1:** Table of sample information. Where relevant median and IQR (in brackets) is given

Parameter	Transplantable (Pass)	Non‐Transplantable (Fail)
Age	52.00 (49.5–56.75)	51.50 (49.75–54.65)
Sex	Five male One female	One male Three female
Donor TLC (L)	6.42 (5.61–7.04)	5.30 (4.82–5.97)
Donor type	Two DCD Four DBD	Four DBD
Smoker	Two Yes Four No	One Yes Three No
Ventilation prior to organ retrieval (days)	2.15 (1.68–3.60)	1.95 (1.33–4.00)
Cold Ischemic time (min)	203.00 (200.50–263.25)	241 (223.25–244.50)
Length of EVLP (min)	161.00 (135.25–178.50)	223.50 (185.25–247.50)
Reason for EVLP assessment failure	N/A	Donor 1 ‐ Poor bronchoscopy with substantial mucosal inflammation, contact bleeding and purulent secretions. Donor 2 ‐ Pulmonary oedema Donor 3 ‐ Pulmonary oedema + inflamed mucosa on bronchoscopy Donor 4 ‐ PaO_2_ < 300 mm Hg + LL atelectasis + poor flow

**FIGURE 1:**
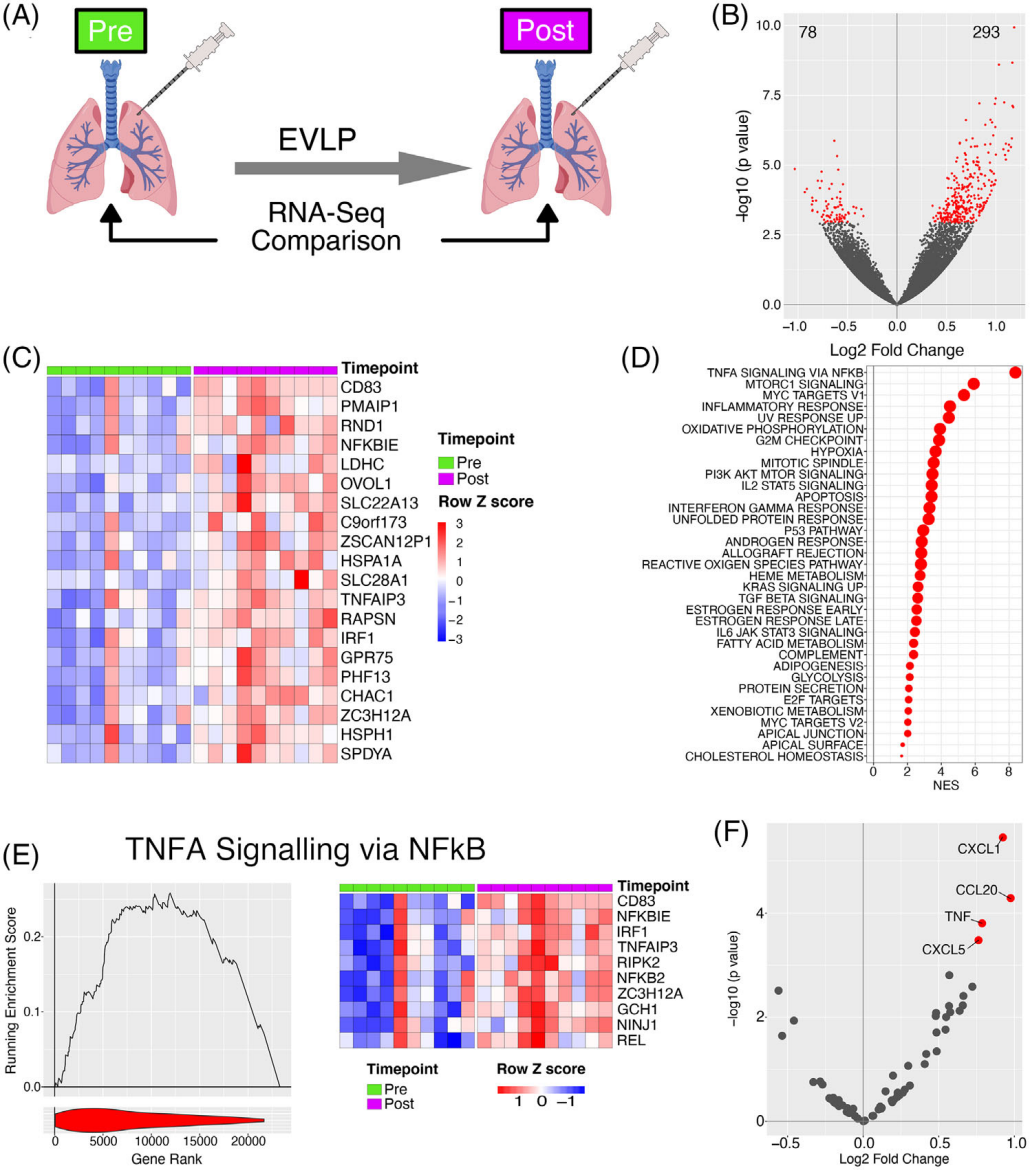
Transcriptional changes occur during EVLP. (A). Diagrammatic representation of the experiment. (B). Volcano plot comparing transcriptome pre and post perfusion. Red points indicate significantly differentially expressed genes (BH adjusted *P* value < .05 and are enumerated on the plot. (C). Heatmap of top 20 significant genes upregulated during EVLP. (D). GSEA analysis against the Hallmarks dataset for EVLP. All significantly enriched pathways (FDR q value < .05) have been plotted, size of point is inversely correlated to the FDR q value, red points indicated positively enriched pathways and blue negatively enriched. (E). Left panel. GSEA enrichment for the TNF via NFkB pathway. Line plot indicates the running enrichment score and violin plot the distribution of genes from the pathways of interest within the ranked gene list. Right panel ‐ top 10 ranked genes within the leading edge genes from the TNFa via NFkB pathway. (F). Volcano plot comparing transcriptome pre and post perfusion showing only cytokine and chemokine genes. Red points indicate significantly differentially expressed genes (BH adjusted *P* value < .05)

We investigated gene expression changes during EVLP by performing RNAseq on pre‐ and post‐EVLP samples (Figure 1A); 293 genes were significantly up‐regulated, and 78 genes significantly down‐regulated during EVLP (Figure 1B, C). Several immune genes were evident in the top 20 up‐regulated genes, including *CD83* and interferon regulatory factor (IRF)1 (Figure 1C). We also observed an increase in components of the NFkB transcription factor complex during EVLP (Figure S1A), as well as *NFKBIE*, a negative regulator of the NFkB pathway (Figure 1C). Heat shock proteins (HSPs) are protein chaperones that contribute to the canonical protective cellular response to a variety of physiological stressors^14^ but also act as innate immune stimulators when released extracellularly.^15^ The HSP70 family proteins *HSPA1A* and *HSPH1* (also known as HSP105) were among the genes most significantly upregulated during EVLP (Figure 1C).

We next assessed changes in the expression of molecular pathways rather than individual expression genes. By considering the net expression of all the genes in a pathway rather than individual genes we are able to better identify patterns in the data whilst also gaining statistical power by reducing the multiple testing burden. Gene set enrichment analysis (GSEA) using gene‐sets curated in the “Hallmarks” database confirmed immune system activation during EVLP, identifying “TNFA signalling via NFkB” pathway as the most enriched in the dataset, as well as enrichment of other immune pathway genes including “Inflammatory response”, and “Interferon gamma response” pathways (Figure 1D, E). Notably, a number of metabolic pathways were also enriched including “Oxidative phosphorylation” and “Fatty acid metabolism” (Figure 1D), both of which contribute to the generation of adenosine triphosphate (ATP), a critical energy source for cells. Together, these data show that during EVLP, a number of immune pathways are induced, as well as pathways involved in energy generation.

We assessed chemokine and cytokine gene expression, and found a significant increase in *TNF*, *CXCL1* and *CXCL5* (neutrophil‐recruiting chemokines), and *CCL20* (a chemoattractant for CCR6‐expressing CD4 T cells and innate lymphoid cells) during EVLP (Figure 1F). At a protein level, CCL2, CXCL8, and ICAM1 in the EVLP perfusate positively correlated with transcript levels in pre‐EVLP biopsies (Figure S1B). In contrast, post‐EVLP transcript levels showed no significant correlation with any perfusate cytokine (Figure S1C). This suggests that proteins released during perfusion reflect pathways already activated prior to EVLP.

### 3.2 Lungs deemed non‐transplantable have increased induction of immune pathway genes following EVLP compared with those deemed transplantable

**FIGURE 2:**
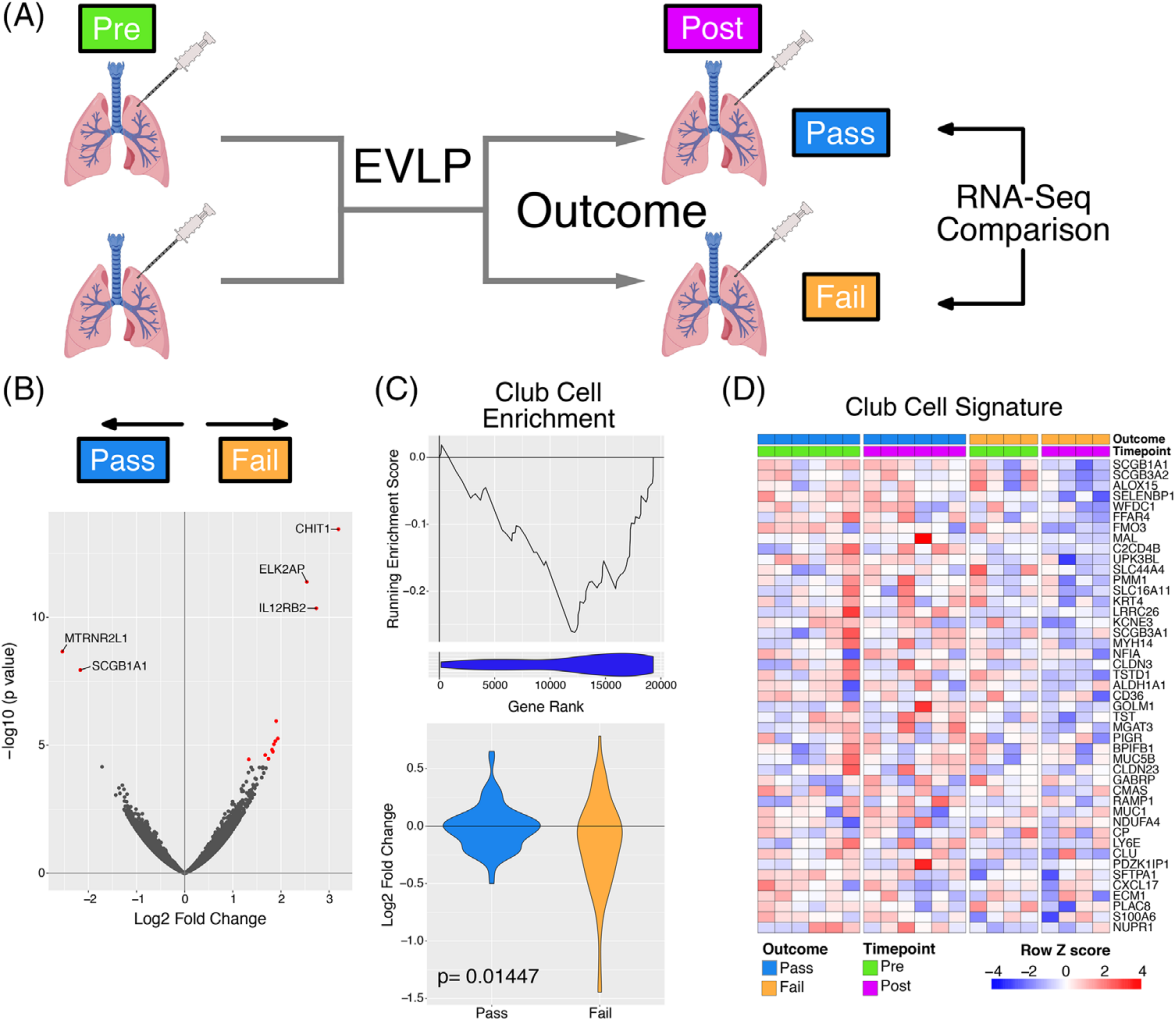
Assessment of differences in the transcriptome following EVLP with respect to outcome. (A). Diagrammatic representation of the analysis. (B). Volcano plot indicating results of differential expression analysis between those lungs which passed and those that failed EVLP post perfusion relative to pass. Red points indicate significantly differentially expressed genes (BH adjusted *P* value < .05). (C). Top panel ‐ GSEA enrichment plot for club cell marker genes from analysis in B. Line plot indicates the running enrichment score and violin plot the distribution of Club cell marker genes within the ranked gene list. Bottom panel – Violin plot indicated the log fold change in expression for all the Club cell marker genes. The *P* value is for comparison of the two groups using a Mann‐Whitney test. Blue is organs which have passed EVLP and orange genes which have failed. (D). Heatmap of leading edge club cell marker genes from B

One of the aims of EVLP is to assess organ function in order to determine suitability for transplantation. Of the *n* = 10 lungs studies, *n* = 6 lungs were deemed transplantable (“Pass”), and *n* = 4 were deemed non‐transplantable (“Fail”) (Table 1, Figure 2A). Comparing the transcriptome of transplantable versus non‐transplantable organs in pre‐EVLP samples, we found no significant differences in gene expression (data not shown). In post‐EVLP samples, 12 genes were significantly upregulated in non‐transplantable (Fail) lungs, including genes of immunological importance, specifically *CHIT1* (encoding chitotriosidase), *CHI3L1* (encoding chitinase‐3‐like 1), *IL12RB2*, *CXCR6*, *SLAMF7, SLC7A5*, and *GBP5* (Figure 2B). CHIT1 is a hydrolase produced by activated alveolar macrophages and has been investigated as a serum biomarker in several lung diseases, including chronic obstructive pulmonary disease.^16^ Chitinase‐like proteins (including CHI3L1) are expressed by alveolar macrophages and play a role in lung inflammation, remodeling and fibrosis.^17^ Taken together, this analysis suggests marked activation of alveolar macrophages in non‐transplantable lungs.

Two genes were significantly upregulated in transplantable lungs, *MTRNR2L1*, (encoding humanin‐like 1, one of a family of proteins that protect cells from oxidative stress^18,^, ^19^), and *SCGB1A1*, a secretoglobin expressed by non‐ciliated bronchiolar epithelial cells and a marker of club (previously known as “Clara”) cells^20^ (Figure 2B). SCGB1A1 (also known as club cell protein 10 (CC10)), is one of several protective proteins produced by club cells.^21^ To further explore their presence in transplantable lungs, we derived a specific transcriptomic signature for club cells from a published lung single cell RNASeq dataset^22^ and found a significant negative enrichment of club cell genes in non‐transplantable (Fail) compared to transplantable (Pass) lungs following perfusion (NES = ‐2.69, FDR q value = 0), with a greater downregulation in fail lungs during perfusion (*P* = .01, Figure 2C‐D, S2). Ciliated epithelial cell and neuroendocrine cell gene signatures were also enriched in transplantable lungs (Figure S2).

**FIGURE 3:**
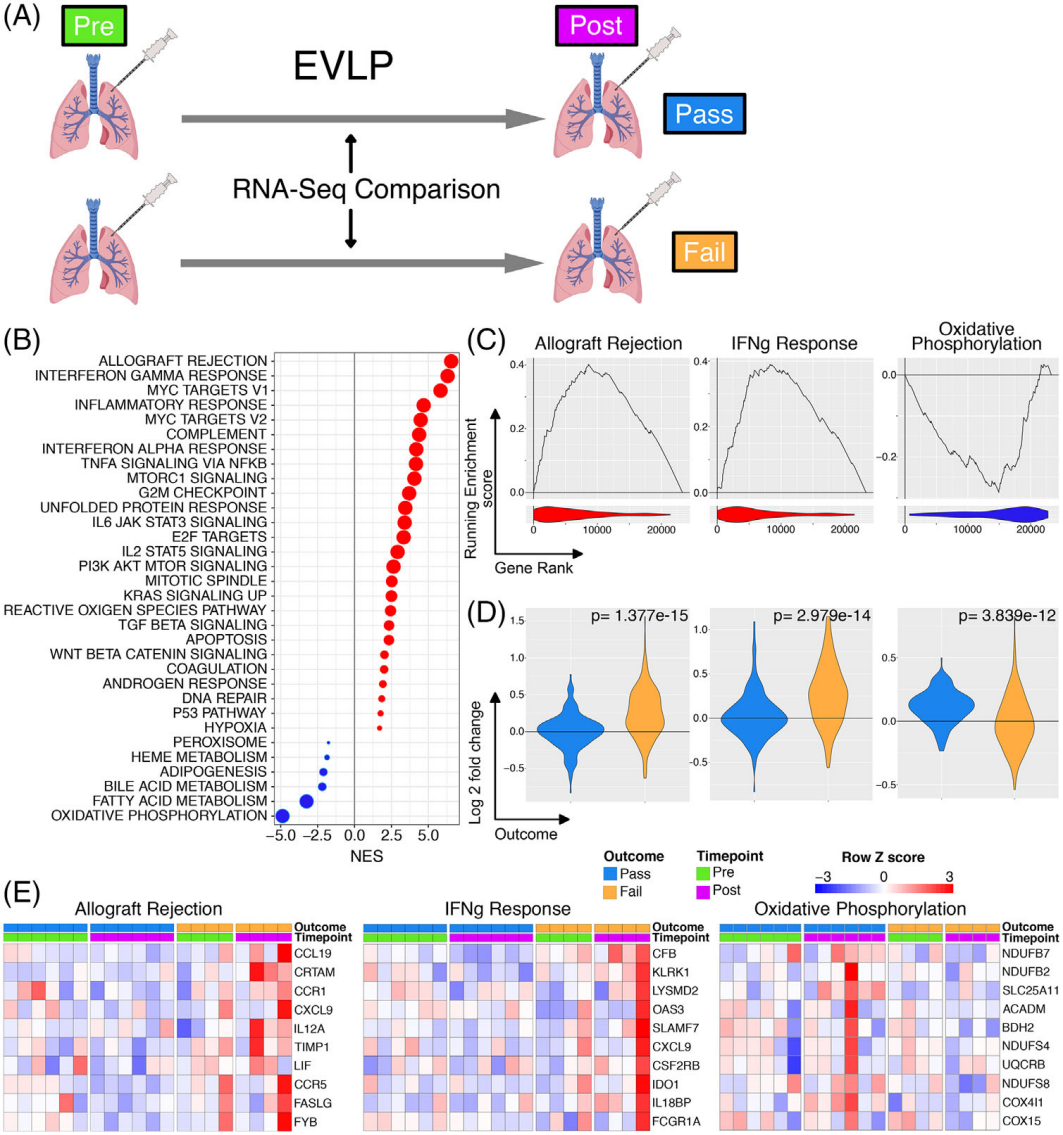
Differential gene expression during EVLP comparing lungs which pass or fail. (A). Diagrammatic representation of the analysis. (B). The interaction of perfusion and outcome was compared and the resulting differential expression data was used to rank genes for GSEA analysis against the Hallmarks database. All significantly enriched pathways (FDR q value < .05) have been plotted, size of point is inversely correlated to the FDR q value, red points indicated positively enriched pathways and blue negatively enriched. (C). Individual enrichment plots from analysis in A. Line plot indicates the running enrichment score and violin plot the distribution of genes from the pathways of interest within the ranked gene list. (D). Transcriptional changes were assessed for pre and post EVLP separately for those organs which passed EVLP verses those that failed. These results were filtered for only the genes in the indicated pathway above each plot and violin plots of log2 fold changes for each gene were plotted. Blue is organs which have passed EVLP, and orange genes which have failed. The *P* value is for comparison of the two groups using a Mann‐Whitney test. (E). Top 10 genes by rank of leading edge genes for the indicated GSEA pathway when comparing the interaction of outcome and perfusion stage

Pathway analysis using GSEA to compare differences between pre‐ and post‐perfusion gene expression in pass and fail lungs (Figure 3A) showed an enrichment of immunological pathways in fail lungs, including “Allograft rejection” and “IFN gamma response” whilst the most negatively enriched pathway, “Oxidative phosphorylation”, showed minimal change during perfusion in those lungs which failed but increased in lungs which passed EVLP assessment (Figure 3B‐E).

### 3.3 Increase in NLRP3 inflammasome‐associated genes in lungs deemed non‐transplantable

**FIGURE 4:**
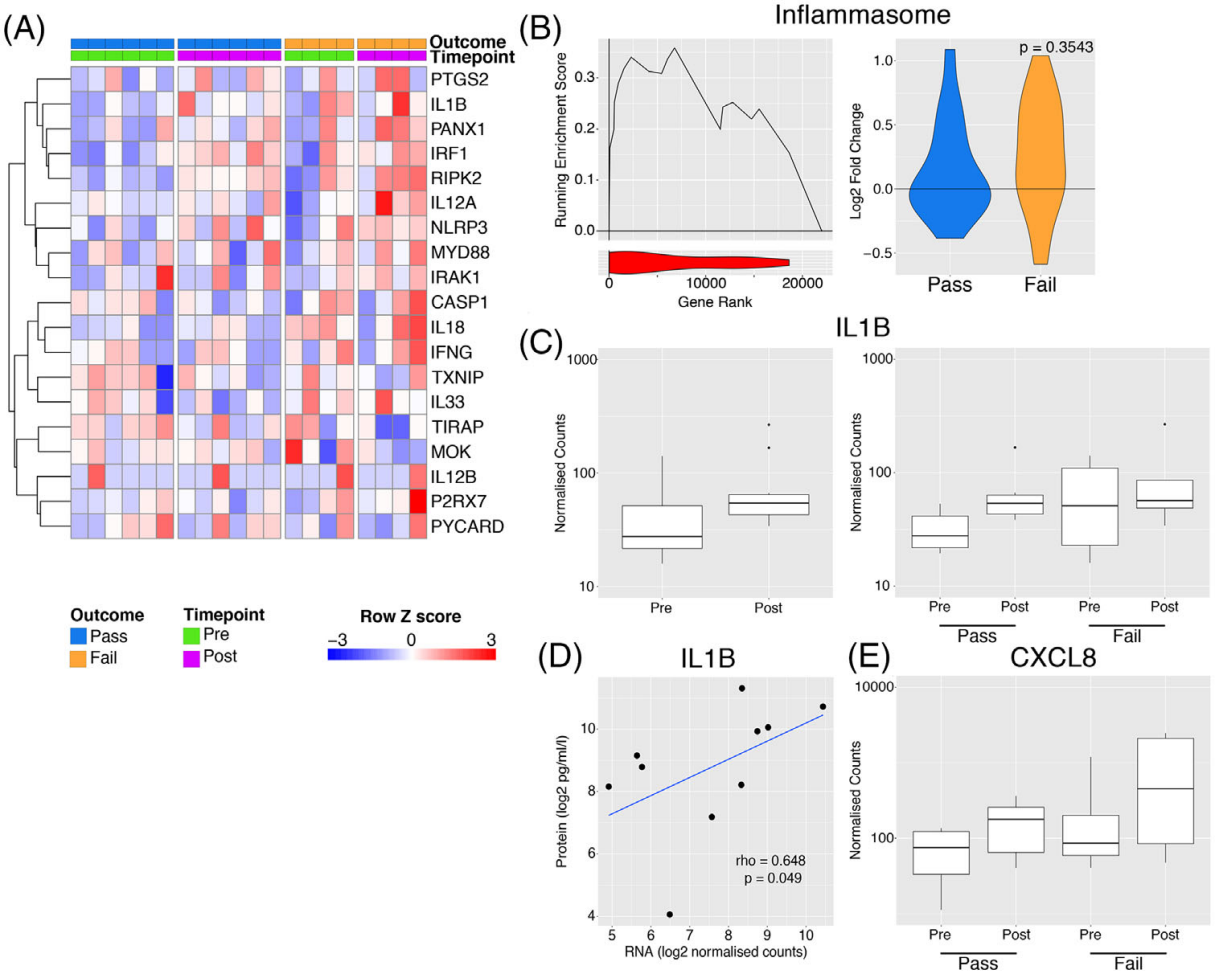
Inflammasome activation with EVLP. (A). Heatmap of genes involved in inflammasome activation. (B). Left panel – GSEA enrichment plot for inflammasome genes from A comparing organs which passed versus those that failed EVLP prior to undergoing perfusion. Right panel – violin plot indicated log2 fold change in inflammasome genes during perfusion separately for the pass (blue) and fail (yellow) groups. The *P* value is for comparison of the two groups using a Mann‐Whitney test. (C). Boxplot of normalized counts for IL1B separated by timepoint alone (left) or both timepoint and outcome (right). (D). Scatter plot of normalized transcript expression prior to EVNP against IL1B protein in the perfusate at the end of EVLP. Correlation coefficient and *P* values was calculated using Spearmans test prior to log2 transformation, blue line indicates a liner model fitted to the data post transformation. (E). Boxplot of normalized counts for CXCL8 in samples pre and post perfusion separated by outcome

Perfusate IL‐1β has been investigated as a predictive biomarker of successful EVLP and post‐transplant outcome in ECD lungs.^23^ The NLRP3 inflammasome acts as a major hub to generate IL‐1β in response to damage‐associated molecular patterns (DAMPs).^24^ Inflammasome associated genes were increased during perfusion, particularly in lungs which failed EVLP assessment compared to those that passed (Figure 4A, B). When considering all 10 lungs analyzed, we found a trend towards an increase in *IL1B* transcripts following EVLP, with non‐transplantable (Fail) lungs showing variable and non‐significantly higher *IL1B* transcripts pre‐EVLP (Figure 4C). *IL1B* gene expression levels in pre‐ (Figure 4D) but not post‐EVLP (data not shown) biopsies significantly correlated with IL1β protein concentration in perfusate at the end of EVLP. Overall, these data suggest that NLRP3 inflammasome activation occurs both prior to and during EVLP, and that this process is more marked in lungs that are deemed non‐transplantable after EVLP.

One effect of IL1β is to stimulate the production of neutrophil‐recruiting chemokines, including CXCL8. There was a non‐significant increase in *CXCL8* transcripts following EVLP, particularly in non‐transplantable lungs (Figure 4E) and variable increases in other neutrophil‐attracting chemokines and adhesion molecules (Figure S3).

### 3.4 Differing patterns of expression of heat shock protein family members during EVLP

**FIGURE 5:**
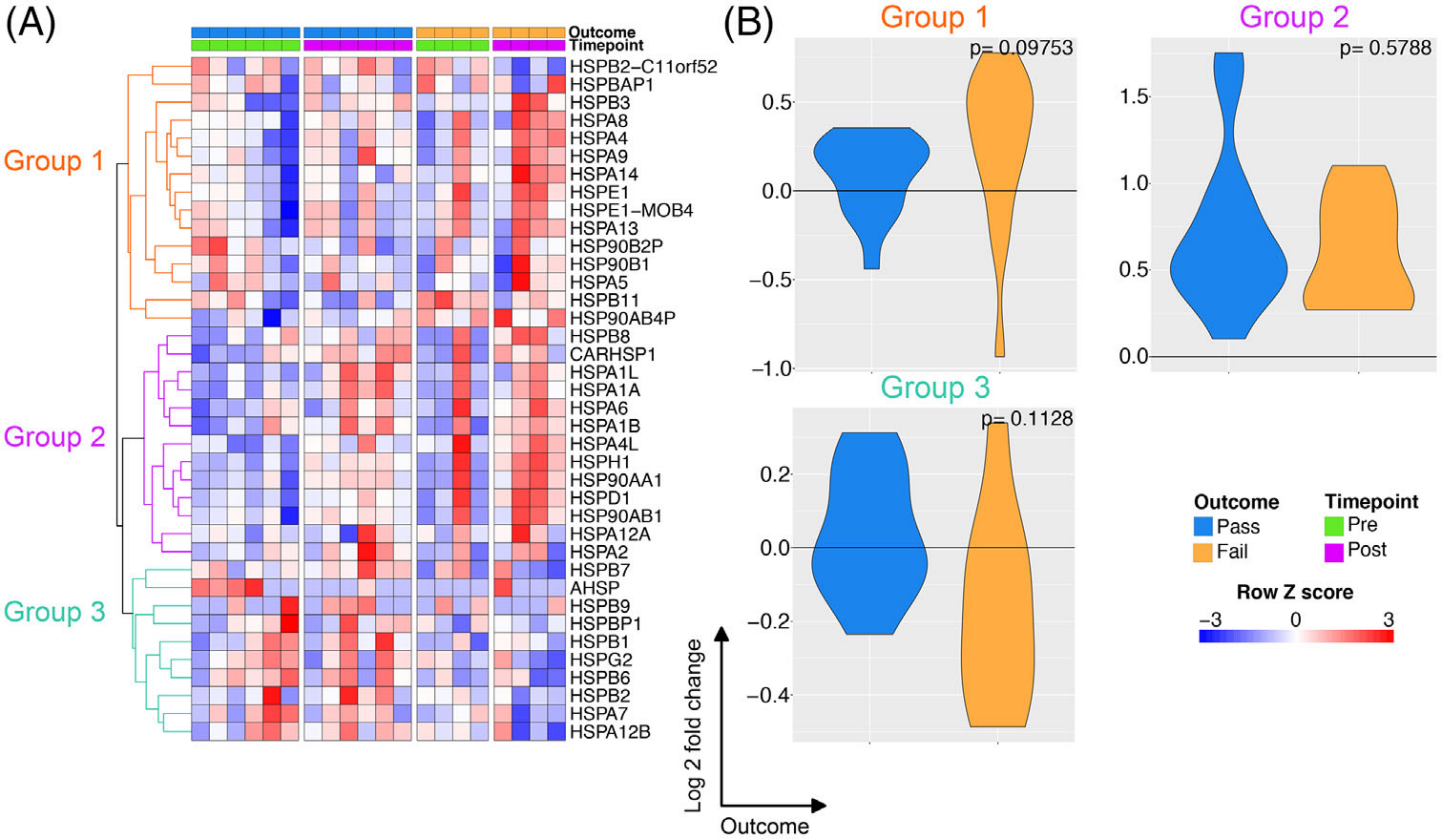
Effect of perfusion on the expression of heat shock protein transcripts. (A). Heatmap of all heat shock protein transcripts in perfusion. The expression profiles were hierarchically clustered and three groups identified using a k‐means approach. (B). Transcriptional changes were assessed for pre and post EVLP separately for those organs which passed EVLP versus those that failed for each group of HSP genes. Blue is organs which have passed EVLP and orange genes which have failed. The *P* value is for comparison of the two groups using a Mann‐Whitney test

Since some HSP70 genes were among those most significantly upregulated during EVLP (Figure 1C), we assessed the expression of all HSP gene family members. This analysis showed three patterns of gene expression in lungs undergoing EVLP: Some HSP family members (gene group 1) were already induced in pre‐EVLP biopsies in both Pass and Fail lungs, and showed little increase in post‐EVLP biopsies in Pass lungs but a more marked increase in Fail lungs (Figure 5A, B), suggesting that they may have deleterious effects. Other HSPs (gene group 2) showed low expression in pre‐EVLP biopsies in both Pass and Fail lungs, and their expression increased in post‐EVLP biopsies in both Pass and Fail lungs (Figure 5A, B). A third group of HSP genes (gene group 3) were already expressed in pre‐EVLP biopsies in both Pass and Fail lungs and their expression was maintained in post‐EVLP biopsies in Pass lungs, but decreased in Fail lungs (Figure 5A, B); suggesting that these may have protective effects. Group 3 HSPs were predominantly HSPB genes, a family of small HSPs.

### 3.5 Assessment of RNA and protein correlates for potential biomarkers

**FIGURE 6:**
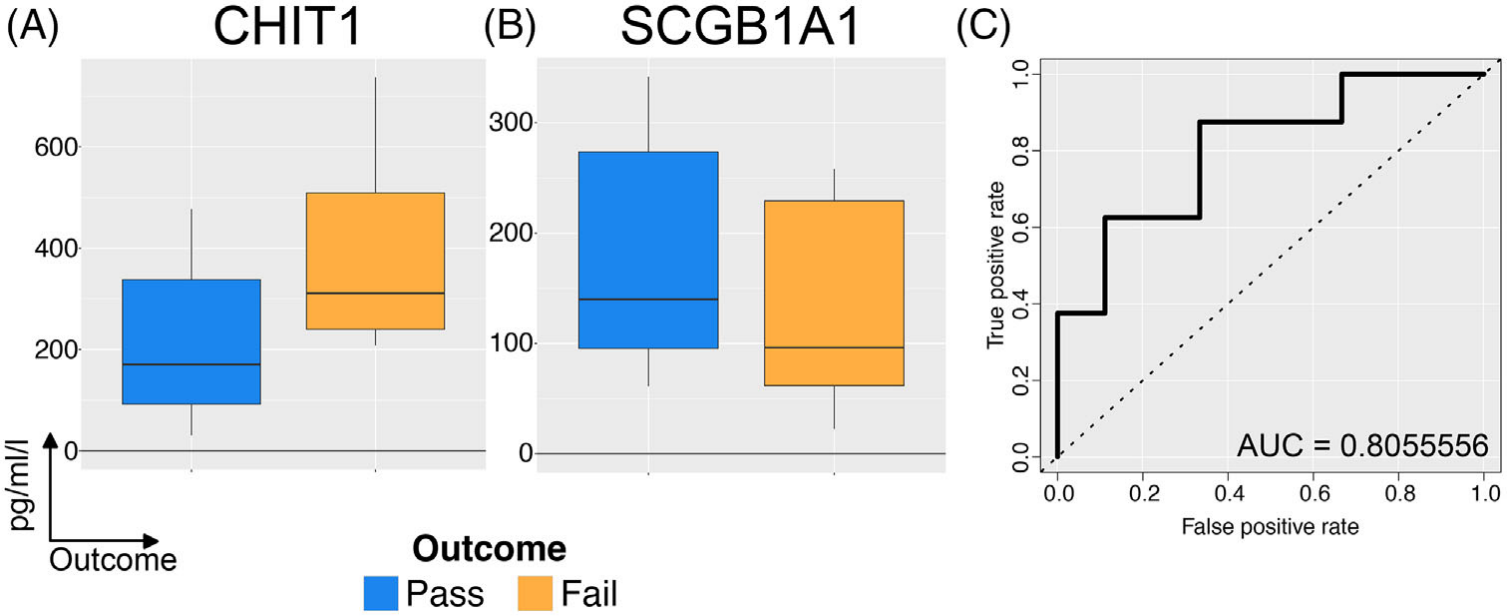
Validation of potential protein level biomarkers for success of perfusion in *n* = 18. (A). Concentration of CHIT1 in perfusate samples taken after 150 min of perfusion with the Lund protocol. Data has been normalized for total lung capacity. (B). Concentration of SCGB1A1 as for A. (C). A ratio of the proteins from A and B was calculated to produce a test statistic which was used for subsequent ROC analysis

We investigated whether the genes differentially expressed in “Pass” and “Fail” lungs (Figure 2A) might be potentially useful biomarkers of successful EVLP. We selected two candidates from our RNASeq data, SCGB1A1 and CHIT1, because they were significantly differentially expressed genes in “Pass” and “Fail” lungs respectively, expressed at a reasonable level, encoded secreted proteins, and quantification reagents/assays were commercially available. Perfusate samples were available on *n* = 17 lungs from the DEVELOP UK study, taken 150 min after the start of perfusion. 7/17 of these lungs were part of the previously described RNASeq study. 9/17 lungs passed EVLP assessment and *n* = 8 failed and were deemed non‐transplantable. We observed an increase in CHIT1 and a decrease in SCGB1A1 in the perfusate of non‐transplantable lungs (Figure 6A). The ratio of these two measurements was a better predictor of EVLP outcome compared to either individually, with a reasonable positive and negative predictive value for lung utilization when applied to a receiver operating characteristic (ROC) analysis (Area under the curve = .8, Figure 6B).

## 4 DISCUSSION

In order for EVLP to be effectively utilized in clinical practice as a platform for the functional assessment, reconditioning and potential treatment of donor lungs, an increased understanding of the cellular and molecular events occurring is required. In this study, we aimed to address this knowledge gap by performing an unbiased analysis of the lung transcriptome prior to and following EVLP. This revealed that several hundred genes were differentially expressed during EVLP, with an induction in a number of innate immune (including *TNF* and *IL1B*) and HSP genes. These data suggest that the tissue damage that occurs during donor lung procurement and hypothermic transport has a substantial capacity to stimulate sterile inflammation during EVLP. Furthermore, the increase in HSPs, (which form part of a canonical cellular stress response), demonstrate the activation of cell‐intrinsic compensatory mechanisms to promote organ viability. HSPs were originally categorized into several families according to their molecular weight, with HSPB family members among those classified as “small” HSPs. Our analysis showed three patterns of HSP gene expression in lungs, and unusable lungs failed to upregulate a subset of HSP genes (dominated by HSPB genes that encode small HSPs). Of note, transgenic over‐expression of the small HSP, HSPB1 (HSP27) has been shown to protect murine hearts and livers from apoptosis in the context of ischemia re‐perfusion injury^25,^, ^26^ and HSPB1 also has anti‐inflammatory effects in airways epithelial cells.^27^ These data suggest that detection of upregulation of this HSP gene subset might be a useful indicator of more suitable lungs that warrants assessment in larger datasets.

Our analysis of post EVLP biopsies identified 12 genes that were statistically significantly upregulated in unusable lungs, and two genes that were upregulated in transplantable lungs. These genes may have potential as novel transcriptional biomarkers but are also likely to reflect functionally significant pathways activated in Pass and Fail lungs. *CHIT1* (encoding chitotriosidase) was the gene most significantly upregulated in unusable lungs. CHIT1 is the most highly expressed chitinase in humans^28^ and is secreted by activated macrophages. Murine models suggest that CHIT1 may enhance TGFβ receptor signaling following lung injury.^29^ Furthermore, in humans, increased *CHIT1* expression has been found in areas of the lung affected by granulomata and fibrosis in tuberculosis, sarcoidosis, idiopathic pulmonary fibrosis, scleroderma, and chronic obstructive lung diseases. Our data raise the possibility that CHIT1 pre‐transplant expression in lungs may be a useful biomarker that identifies donor lungs likely to perform poorly during EVLP.

*CHI3L1* was also significantly upregulated in unusable lungs. Chitinase‐like proteins are evolutionarily conserved in mammals and appear to have evolved to play a role in the development and progression of Th2 immune responses and in defense against parasitic infections and cancer.^30,^, ^31^ Chitinase‐like proteins are expressed by macrophages and T cells and are involved in lung inflammation, remodeling, and fibrosis^17^ and *CHIT1* and *CHI3L1* have previously been shown to be induced in alveolar macrophages during mycobacterial infection.^32^ Other genes upregulated in non‐transplantable lungs included *SLC7A5* and *GBP5*. SLC7A5 is an amino acid transporter that mediates lysine influx and contributes to IL1β production via mTOR complex 1 (mTORC1)‐induced glycolytic reprograming of activated human monocytes^33^ whilst GBP5 promotes selective NLRP3 inflammasome assembly in macrophages in response to soluble stimuli.^34^ Taken together, our data suggest that macrophage activation is more marked in unusable lungs compared with those deemed transplantable. Whether this is due to higher levels of tissue damage and associated DAMPs, or due to a higher macrophage‐intrinsic capacity to respond to DAMPs remains to be determined.

Two genes were significantly upregulated in transplantable lungs, MTRNR2L1, (encoding humanin‐like 1), and SCGB1A1, a secretoglobin expressed by club cells.^20^ The humanin gene family encodes for small secretory peptides^35^ that have been shown to protect cells from oxidative stress, starvation and hypoxia^19^ and MTRNR2L1 transcription is increased by oxidative stress and toll‐like receptor 4 agonists,^36^ therefore, its upregulation may enhance cellular integrity in lungs in the face of cell stressors associated with the organ procurement process. Club cells are the major secretory cells of the human small airways that have anti‐protease activity via the secretion of α1‐antitrypsin (an important neutrophil elastase inhibitor) and SLPI (secretory leucoprotease inhibitor, a potent serine protease inhibitor).^20^ SCGB1A1 has been shown to have direct anti‐inflammatory actions inhibiting NFkB activation and CXCL8 secretion in airway epithelial cells.^21,^, ^37^ Our data raises the possibility that club cells function to protect lungs from some of the deleterious processes occurring during the organ donation and procurement process and to inhibit collateral damage caused by neutrophils recruited following reperfusion. It will be possible to test this hypothesis in future studies by investigating club cell number and activation in pre‐implantation biopsies in a larger cohort of transplanted lungs.

The major limitation of our study is the relatively small sample size (*n* = 10) but this is not dissimilar to other transcriptomic studies in lung transplantation.^38,^, ^39^ However, our use of paired pre‐ and post‐EVLP samples, as well as gene pathway analysis, substantially increased our statistical power to demonstrate meaningful increases in functionally relevant molecular pathways. Our study identified a number of novel RNA biomarkers that may indicate successful EVLP and we went on to measure the protein concentration of two of these biomarkers, CHIT1 and SCGB1A1, in perfusate in a larger validation cohort. This analysis indicated that these two measurements can be combined to predict successful EVLP with reasonable sensitivity and specificity, and merit prospective assessment in future clinical trials. Our results also suggest a number of potential targets for therapeutic intervention to improve lung suitability during EVLP that will require validation in larger studies. In particular, inhibition of the NLRP3 inflammasome and enhancement of club cell function may be of utility. The former is readily translatable, given the development of these agents for the treatment of sterile inflammation in other contexts, such as crystal‐induced arthropathies and autoinflammatory conditions.^40^

In summary, our data suggest that lungs deemed suitable for transplantation following EVLP have reduced induction of multiple immune pathways and are better able to generate ATP. Our study identifies specific biomarkers that may hold utility in identifying suitable lungs and pathways amenable to therapeutic intervention during EVLP that will inform the design of future clinical trials in this area.

## CONFLICTS OF INTEREST

None of the authors have any finical conflicts of interest to disclose.

## AUTHOR CONTRIBUTIONS

J.R.F., M.M., A.J.F., and M.R.C. conceived and designed the research project. J.R.F., M.M., C.C., A.C., and L.E.B. carried out experiments. A.A. and W.E.S. collected, processed and stored samples. J.R.F. analyzed the data. J.R.F. and M.R.C. interpreted the data. M.M., J.R.F., and M.R.C. wrote the manuscript, and A.J.F. edited the manuscript. All authors reviewed and commented on the manuscript.

## Supporting information

Supporting Information

## Data Availability

Data will be made available following all reasonable requests to the corresponding author.
