# Supplementary material for: Transcriptional analysis identifies potential novel biomarkers associated with successful ex‐vivo perfusion of human donor lungs
Source: Clin Transplant. 2022 Jan 10;36(4):e14570. doi: 10.1111/ctr.14570 (PMC9285052; doi:10.1111/ctr.14570)
Supplement: Supplementary file 1 — Supporting Information [file CTR-36-0-s001.pdf]

Title: Transcriptional analysis identifies novel biomarkers associated with successful ex-vivo perfusion of human donor lungs

Authors:

John R. Ferdinand, Morvern I. Morrison, Anders Andreasson …

Supplementary Appendix

Table of contents

Supplementary methods --- pages 2-3

Supplementary figures --- pages 4-8

Supplementary Methods

scRNAseq analysis

The dataset GSE103354 was used to produce a club cell specific gene signature (*39*). Briefly the publicly available data was analysed using the standard Seurat pipeline. Following normalisation and scaling of the data clusters were identified and annotated. Marker genes for the club cell cluster were determined and the top 100 as ranked by log fold change were used as the Club cell specific gene set for subsequent analysis.

Gene Set Enrichment Analysis

For GSEA comparisons of interest were ranked by the inverse of the raw p value with the sign of the log fold change such that the gene ranked 1 showed the most significant positive fold change and the nth gene the most significant negative fold change. Genes which showed no difference are centred around nth/2 rank. GSEA was subsequently carried out using the java based GSEA applet maintained by the Broad using the classic enrichment statistic in pre-ranked mode.

Soluble protein measurements

Serial perfusate samples from 17 human donor lungs undergoing clinical EVLP assessments were analysed retrospectively. Interleukin (IL)-1b, IL-6, IL-8, TNF-a, and MCP-1 were analysed with an MSD Multi-ArrayVR (Meso Scale Diagnostics, LLC, Rockville, MD, USA). The assay was performed according to the manufacturer's instructions. Enzyme Linked Immunosorbent Assays (ELISA) were used to detect protein levels in the perfusate for CHIT1 (BioTeche Novus Biologicals, Abingdon, Oxfordshire, UK), SCGB1A1 and soluble Intercellular Adhesion Molecule 1 (ICAM-1) (R&D Systems, Inc., Minneapolis, MN, USA). All

protein expression measured in perfusate was adjusted to the predicted total lung capacity (pTLC) of the donor as an estimate of perfused donor lung volume and were reported as corrected perfusate concentrations (pg/ml) by dividing the concentration in the perfusate by the pTLC. The pTLC was calculated in a routine fashion based on donor gender and height.

Receiver Operating Characteristic Analysis

A test statistic for prediction of EVLP outcome was produced by taking the ratio of the concentration of CHIT1 to SCGB1A1 protein present in perfusate at 150 min post perfusion from 17 perfused lungs made up from the DEVELOP UK study including 7 from the set of samples used for the RNAseq analysis and 10 from further lungs within the study but not included in the RNAseq analysy. The R packaged ROCR was used to perform the test and an area under the curve was calculated.

Figure generation

Some of the illustrations contain images produced using ©BioRender.

Data availability

Original Data will be made available upon all reasonable requests to the corresponding author.

Supplementary figures

Supplemental Figure 1

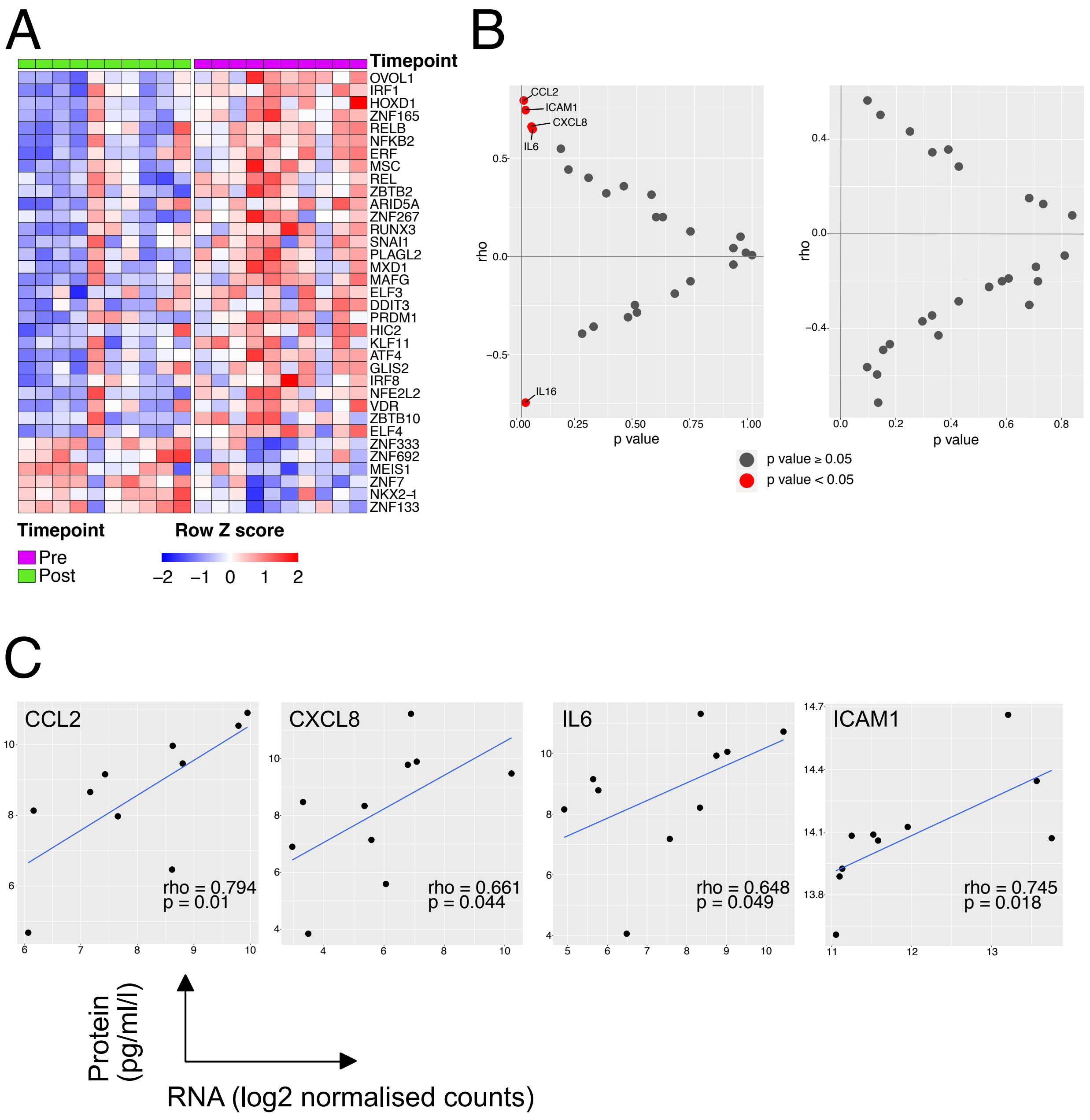

Supplemental Figure 1 - A – Heatmap of all genes identified as transcription factors in the TFCheckpoint database and significantly differentially expressed during EVLP **B** - Correlation of transcriptome either pre (Left) or post (Right) EVLP with cytokines in the perfusate at the end of the procedure. Cytokines were measured in perfusate using a multiplex MSD kit. The normalised RNA level in the samples prior to EVLP was correlated using Spearmans method

with the protein level detected in the perfusate. Correlations with a p value of less than 0.05 have been labelled. **C**- Scatter plot of normalised transcript expression prior to EVNP against protein level in the perfusate at the end of EVLP. Correlation coefficient and p values was calculated using Spearmans test prior to log2 transformation, blue line indicated a liner model fitted to the data post transformation. Cytokines compared are indicated at the top of the respective plot.

Supplemental Figure 2

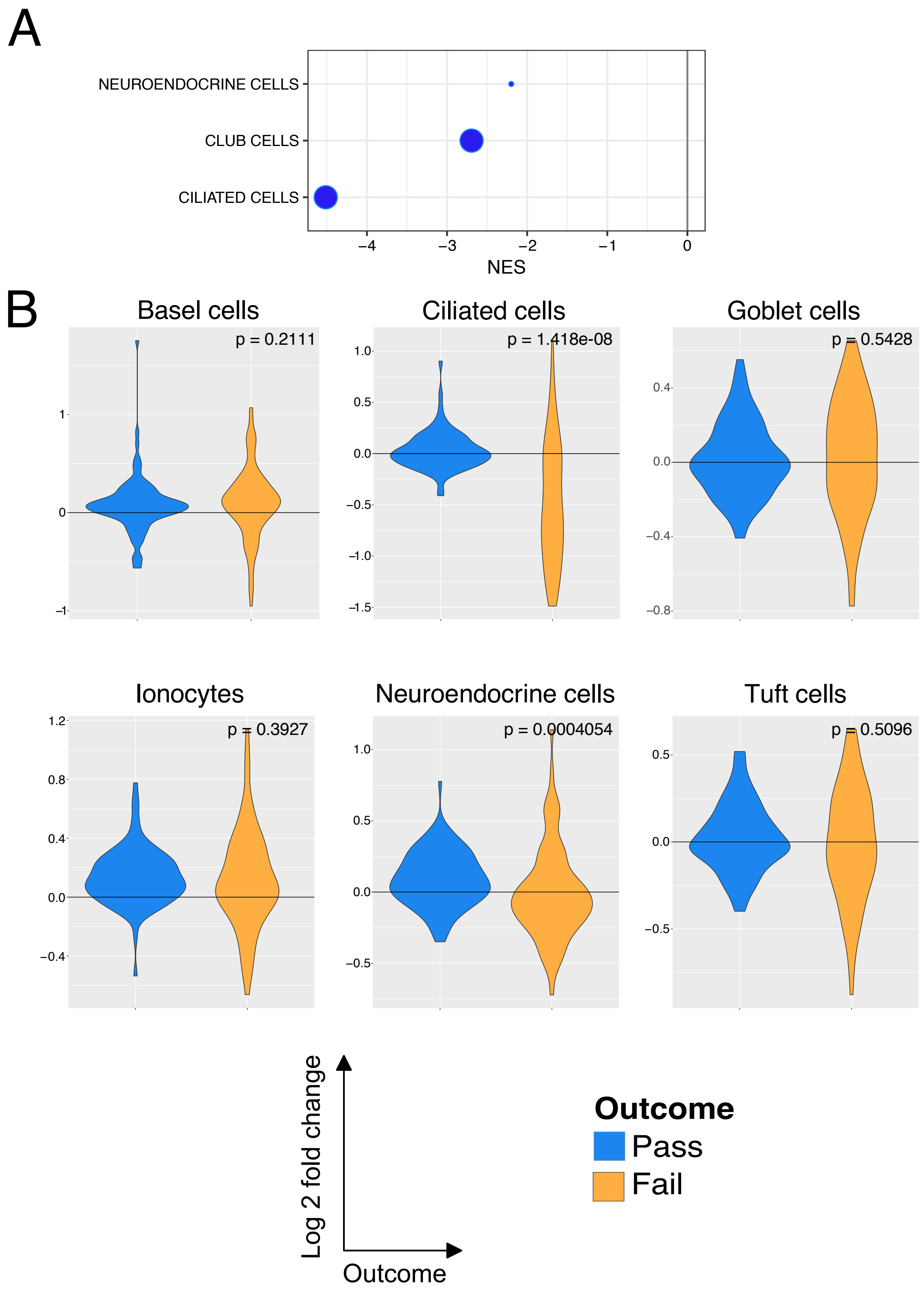

Supplemental Figure 2 – **A** – GSEA analysis for all lung cell subset marker genes following EVLP comparing those lungs which passed and those that failed EVLP. All significantly enriched pathways have been plotted, size of point is inversely correlated to the FDR q value, red points indicated positively enriched pathways and blue negatively enriched. **B** - Violin plot indicated the log fold change in expression for all lung cell subset marker genes. The p value is for comparison of the two groups using a Mann-Whitney test. Blue is organs which have passed EVLP and orange genes which have failed.

Supplemental Figure 3

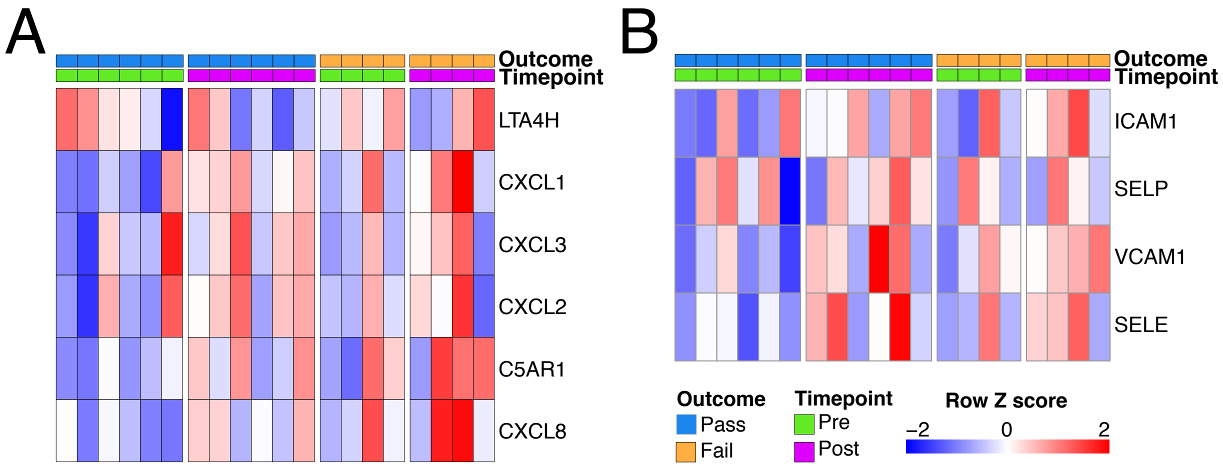

Supplemental Figure 3 – Neutrophil modulation during EVLP. **A** - Heatmap of genes involved in neutrophil chemoattraction. **B** - Heatmap of genes involved in neutrophil adhesion.
